# Supplementary material for: Chromothripsis during telomere crisis is independent of NHEJ, and consistent with a replicative origin
Source: Genome Res. 2019 May;29(5):737–49. doi: 10.1101/gr.240705.118 (PMC6499312; doi:10.1101/gr.240705.118)
Supplement: Supplemental Material [file supp_gr.240705.118_Supplemental_file_1.zip › contigs/annotated_contigs/DB51/contig.2.DB51_length_569_mean_cov_8.53427065026.docx]

**DB51_length_569_mean_cov_8.53427065026**

```
ATTTCTCCCAGTGGCTTCATTAGTTTTTCCATGTTTTAAGAACTCTATAGCTACATTTCCTAAGAGACATTTATTTCTGTGTTTCCTAG
 >chr6:82677837-82678049 + E=7e-116
TTATAGGTTTATAGATTACTAGGAGTTCTGGGTCACTGTGTTACTATAAGATTGAGTTCCAACTTTATGACTCATTTTCTAAAAATCAA

TTTTCTCAAGCCACTGTCCATTTAATTTCATTGG|AACATATGGAAAGCAGTCAACAGGATATTCATTAAGTCTAGAAACACAGATGGG
                                    >chr6:10514974-10515331 - E=3e-200
TATACATAACTTCATCAAGACCATTTTCAATGTGTAACACACACGTGTGCGTGCACTCACAGAGTTTAGACTTAATGTAGATTGGAAAT

CAAGTTCCACATACCTGGAATCCTACTGAGTGTCACCCAGAAATGCTCATCAGGAGAGTCAGTATCTTTTGACCACTCAAGTAAATCAA

TGGCCCTTAGGTTTTGAAGAACAAAATTAGCAAATTCCCTTGTAAGGGCCACATAGGCAGAGCCAAAGTAAATTGTCAGATGGTGGGGA

GGAGGCATTTTTCTCATGCAAGTCCACAGCATGAAG
```
